# Lanthanide Molecular Species Generated Fe_3_O_4_@SiO_2_-TbDPA Nanosphere for the Efficient Determination of Nitrite

**DOI:** 10.3390/molecules27144431

**Published:** 2022-07-11

**Authors:** Xiangqian Li, Qin Wen, Jiannian Chen, Wenjie Sun, Yuhui Zheng, Chenggang Long, Qianming Wang

**Affiliations:** 1 Key Lab of Ecological Restoration in Hilly Areas, School of Chemical & Environmental Engineering, Pingdingshan University, Pingdingshan 467000, China; lixq605@163.com; 2 Guangzhou Key Laboratory of Analytical Chemistry for Biomedicine, School of Chemistry, South China Normal University, Guangzhou 510006, China; a18925131809@163.com (J.C.); 13842933314@163.com (W.S.); yhzheng78@scnu.edu.cn (Y.Z.); 3 Institute of Biomedical Engineering, College of Life Sciences, Qingdao University, Qingdao 266071, China; qwen@qdu.edu.cn; 4 Ruide Technologies (Foshan) Inc., Foshan 528311, China; hychen7788@126.com

**Keywords:** nitrite, core-shell nanospheres, fluorescent probe

## Abstract

The presence of nitrite (NO_2_^−^) in water and food leads to serious problems in public health and the environment. Therefore, it is important to develop a rapid and efficient method for the selective detection of NO_2_^−^. In this work, the synthesis and characterization of magnetic Fe_3_O_4_@SiO_2_-TbDPA nanoprobe have been carried out. The Fe_3_O_4_@SiO_2_-TbDPA aqueous solution exhibits a strong green emission. Due to the addition of various concentrations of NO_2_^−^ (0–100 μM), the fluorescence intensity has been suppressed. The nanoprobe Fe_3_O_4_@SiO_2_-TbDPA exhibits excellent selectivity and sensitivity toward NO_2_^−^ ions. Excellent linearity is obtained in the range of 5–80 μM with a detection limit of 1.03 μM. Furthermore, the presence of magnetic Fe_3_O_4_ nanoparticles in Fe_3_O_4_@SiO_2_-TbDPA nanospheres will also facilitate the effective separation of Fe_3_O_4_@SiO_2_-TbDPA from the aqueous solution. Our proposed strategy is expected to fabricate an organic-inorganic hybrid magnetic nanomaterial and can be used as an efficient sensor. It has been shown that this new strategy has numerous advantages, such as high stability, selectivity, and simplicity of operation. It demonstrates great potential for simple and convenient NO_2_^−^ detection. It may expand to a variety of ranges in environmental monitoring and biomedical fields.

## 1. Introduction

Nitrite (NO_2_^−^) is an important substance that is widely distributed in the environment and food. It has been extensively used in meat preservation and processing in order to inhibit the propagation of Clostridium botulinum and to improve the flavor of meat [1]. Meanwhile, NO_2_^−^ is a well-known signaling molecule that plays a vital role in normal physiological activities, such as hypoxia, nitric oxide homeostasis and bloodstream regulation [2]. However, excessive intake of NO_2_^−^ can lead to a variety of disorders, including intrauterine spontaneous abortion, growth retardation, central nervous system congenital defects and infant methemoglobinemia [3,4]. Furthermore, the presence of NO_2_^−^ ions in synthetic urine can be employed as an indicator of urinary tract infections [5]. Because of the toxicity of NO_2_^−^ ions, the accepted maximum contaminant levels (MCL) of NO_2_^−^ ions in drinking water are regulated to be 214.2 μM by the World Health Organization (WHO) and 71.4 μM by the U.S. Environmental Protection Agency (EPA) [6]. Traditional methods for the detection of NO_2_^−^ are given as spectrophotometry, high-performance liquid chromatography, ion chromatography, gas chromatography, electrophoresis, electrochemical methods and so on [7,8,9,10,11]. Although these methods have considerable sensitivity and detection limits, most of them require complex equipment and instruments and tedious operational procedures and skills, which lack realistic operability and make it difficult to achieve highly sensitive, selective, qualitative and quantitative detection of NO_2_^−^ [12,13,14]. Fortunately, fluorescent probes have become a suitable tool for NO_2_^−^ detection due to their easy and rapid operation, highly sensitive, highly selective, and low cost [15,16,17]. Therefore, the realization of preparation for efficient and reliable determination of NO_2_^−^ will be expected.

Lanthanide ions exhibit specific luminescence properties, including high quantum efficiency, extraordinary color purity, a long lifetime, sharp emission peaks, and large Stokes shifts [18,19,20]. Due to these merits, lanthanide luminescent materials have been widely studied in chemical and biological sensing systems [21,22,23]. In recent years, the development of lanthanide luminescent fluorescent probes for guest molecule (e.g., anions, cations, biomolecules) recognition has become an emerging field [24,25,26,27]. Our group has been devoted to the preparation of lanthanide hybrid materials and sensing applications, and the rapid development for the achievement of specific lanthanide sensors has been reported [28,29,30]. Previously, we found a ClO^−^ highly selective Europium sensor based on an oxime isomerization reaction for the first time [31]. Magnetic nanomaterials are one of the most important classes of materials due to their superior properties and their wide application in science and technology [32,33]. Taking these into account, we believe that the grafting of lanthanide ions onto magnetic nanomaterials will induce effective results. At present, this has never been mentioned for the smart magnetic nanomaterial in response to NO_2_^−^ ions.

**Figure 1:**
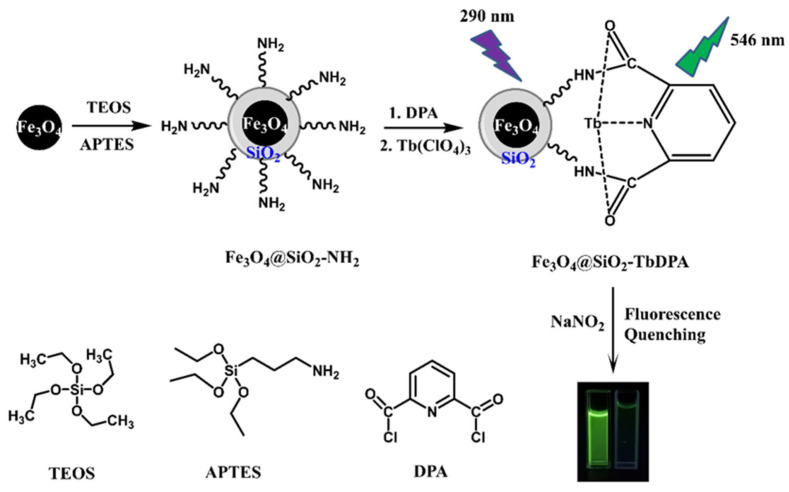
Schematic diagram of Fe_3_O_4_@SiO_2_-TbDPA nanoprobe for NO_2_^−^ detection.

In this study, we rationally designed and fabricated a selective and sensitive fluorescent nanoprobe, Fe_3_O_4_@SiO_2_-TbDPA, and its effective detection of NO_2_^−^ in the water sample was explored (Figure 1). The as-prepared Fe_3_O_4_@SiO_2_-TbDPA was fully investigated by FT-IR, UV-Vis, TEM, SEM and fluorescence spectroscopy. Fe_3_O_4_@SiO_2_-TbDPA aqueous solution exhibits a strong green emission. Its fluorescent signal was decreased depending on the presence of NO_2_^−^. Fe_3_O_4_@SiO_2_-TbDPA exhibited good selectivity and sensitivity toward NO_2_^−^ in a 100% aqueous solution. Moreover, after detection, the magnetic-sensitive nanoprobe was separated from the aqueous solution by taking advantage of their magnetic properties. The results shed new lights on the determination process in practical environments and biomedical analysis.

## 2. Experimental Section

### 2.1. Reagents and Materials

Terbium perchlorate (Tb(ClO_4_)_3_·6H_2_O) was acquired from the Shanghai Yuelong company (Shanghai, China). Ferroferric oxide (II, III) (25% in H_2_O), ammonia solution (NH_3_·H_2_O, 25%), tetraethyl orthosilicate (TEOS, 99.9%) (SiC_8_H_20_O_4_), aminopropyltriethoxysilane (APTES, 99.9%) (C_9_H_23_NO_3_Si), Triton X-100, 1-hexanol and 2,6-pyridinedicarboxylic acid chloride were purchased from Aladdin Chemistry Co. Ltd. (Shanghai, China). All the other metal salts and reagents were purchased from Guangzhou Chemical Reagent Factory (Guangzhou, China) and used without second purification.

### 2.2. Characterization

Transmission electron microscope (TEM) images were obtained with a JEOL JEM-2100 HR transmission electron microscope. Scanning electron microscopy (SEM) images were measured by a Tescan 5136MM scanning electron microscope. FT-IR spectra of the materials were measured within the 4000–400 cm^−1^ wavenumber range by using a Prestige-21 spectrometer. UV-vis spectra were recorded on an Agilent 8453 UV-visible spectrophotometer. The magnetic properties of the samples were measured at room temperature using a BHV-55 vibration sample magnetometer (VSM) with an applied field of −5000–5000 Oe. The fluorescence spectra were collected with a Hitachi-4600 fluorescence spectrophotometer.

### 2.3. Preparation of Fe_3_O_4_@SiO_2_-NH_2_ Nanospheres

The Fe_3_O_4_@SiO_2_-NH_2_ nanospheres were prepared according to the literature procedure with slight modifications [33]. Briefly, 0.13 g Fe_3_O_4_, 18 g Triton X-100, 16 mL n-hexanol and 75 mL cyclohexane were mixed in a 250 mL glass flask with vigorous mechanical agitation; then, 4 mL deionized H_2_O was immediately added. Subsequently, 1 mL TEOS was added to the above solution, and the mixture was stirred for 30 min. Lastly, 0.7 mL NH_3_·H_2_O was added to the above mixture to initiate silica polymerization, and the polymerization was allowed to proceed for 18 h. The resulting Fe_3_O_4_@SiO_2_-NH_2_ nanospheres were washed with water and ethanol three times and then magnetically separated using a simple bar magnet. The as-prepared Fe_3_O_4_@SiO_2_ nanospheres were redispersed into a 20 mL ethanol solution. Then, 0.5 mL APTES and 0.3 mL NH_3_·H_2_O were added. This suspension was stirred at room temperature for 12 h. The resulting Fe_3_O_4_@SiO_2_-NH_2_ nanospheres were collected by magnetic decantation and purified by ethanol via repeated washing. Finally, the Fe_3_O_4_@SiO_2_-NH_2_ magnetic nanospheres were dried under vacuum at 60 °C for 12 h.

### 2.4. Preparation of Fe_3_O_4_@SiO_2_-DPA Nanospheres

In order to obtain the 2,6-Pyridinedicarboxylic acid chloride-modified Fe_3_O_4_@SiO_2_, the prepared Fe_3_O_4_@SiO_2_-NH_2_ nanospheres (0.10 g) and 2,6-pyridinedicarboxylic acid chloride (0.30 g) were suspended in anhydrous toluene (20 mL) and refluxed for 10 h under an N_2_ atmosphere. The obtained functionalized Fe_3_O_4_@SiO_2_-DPA nanospheres were washed three times with ethyl alcohol to remove excess 2,6-pyridinedicarboxylic acid chloride and then dried under vacuum at 60 °C for 12 h.

### 2.5. Fabrication of Terbium Hybrid Materials (Fe_3_O_4_@SiO_2_-TbDPA)

A total of 50 mg Fe_3_O_4_@SiO_2_-DPA and 50 mg Tb(ClO_4_)_3_·6H_2_O were dispersed in 20 mL ethanol. Then, 0.2 mL NH_3_·H_2_O was added, and the mixture was refluxed for 8 h. After centrifugation, the precipitate was washed with ethanol three times and dried under vacuum at 60 °C for 12 h to yield Fe_3_O_4_@SiO_2_-TbDPA nanospheres.

### 2.6. Optical Studies

The stock solution of 0.1 mg/mL Fe_3_O_4_@SiO_2_-TbDPA and 10 mM (Na_2_CO_3_, Na_2_SO_4_, Na_2_HPO_4_, NaH_2_PO_4_, CH_3_COONa, NaNO_3_, NaF, NaCl, NaBr, NaI and NaNO_2_) were prepared in deionized water, respectively. Fluorescence response of Fe_3_O_4_@SiO_2_-TbDPA toward different anions was performed by introducing 100 μM (CO_3_^2−^, SO_4_^2−^, HPO_4_^2−^, H_2_PO_4_^−^, CH_3_COO^−^, NO_3_^−^, F^−^, Cl^−^, Br^−^, I^−^ and NO_2_^−^) into Fe_3_O_4_@SiO_2_-TbDPA (0.1 mg) aqueous solution at room temperature, respectively.

## 3. Results and Discussion

### 3.1. FT-IR Analysis

**Figure 2:**
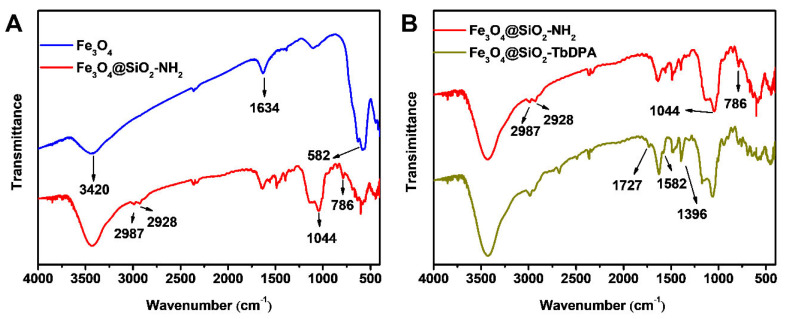
FT-IR of Fe_3_O_4_, Fe_3_O_4_@SiO_2_-NH_2_ (**A**) and Fe_3_O_4_@SiO_2_-TbDPA (**B**).

The surface functional groups of Fe_3_O_4_, Fe_3_O_4_@SiO_2_-NH_2_ and Fe_3_O_4_@SiO_2_-TbDPA nanospheres were studied using the FT-IR technique. As shown in Figure 2A, the strong, broad peak at about 582 cm^−1^ was attributed to the stretching vibration of the Fe-O bond, indicating the formation of magnetic Fe_3_O_4_ nanoparticles [34,35,36]. The broad bands at 1634 cm^−1^ and 3420 cm^−1^ were attributed to the O-H bending and stretching vibrations of water molecules [37].

After surface modification, Fe_3_O_4_@SiO_2_-NH_2_ nanospheres possessed absorption bands caused by symmetric vibration of Si-O-Si (786 cm^−1^) and asymmetric vibration of Si-O-Si (1044 cm^−1^) [26]. The emerging absorption bands at about 2928 cm^−1^ and 2987 cm^−1^ were attributed to the stretching vibrations of –CH_2_– groups from APTES units. The results supported that the SiO_2_-NH_2_ layer has covered the surface of the Fe_3_O_4_ nanoparticle. The appearance of two bands of 1727 and 1396 cm^−1^ in Fe_3_O_4_@SiO_2_-TbDPA corresponded to C=O-NH and the stretching vibration of C=O [38]. The weak absorption peak at 1582 cm^−1^ was assigned to the pyridine ring. The collected results indicated that the DPA molecule was successfully grafted onto the outer surface of Fe_3_O_4_@SiO_2_-NH_2_ and coordinated with a terbium ion to form Fe_3_O_4_@SiO_2_-DPA hybrid materials (Figure 2B).

### 3.2. UV-Vis Analysis

To further evaluate the structural information of Fe_3_O_4_@SiO_2_-TbDPA nanospheres, analyses of the UV-Vis spectra of Fe_3_O_4_, Fe_3_O_4_@SiO_2_-NH_2_ and Fe_3_O_4_@SiO_2_-TbDPA were carried out (Figure S1). Both Fe_3_O_4_ and Fe_3_O_4_@SiO_2_-NH_2_ suspension displayed a weak absorption at 383 nm, while Fe_3_O_4_@SiO_2_-TbDPA in water gave rise to not only the band at 383 nm but also the new signals at 270 and 279 nm. The achieved bands were derived from 2,6-pyridinedicarboxylic acid chloride. These results verified the successful modification of the organic ligands 2,6-pyridinedicarboxylic acid chloride onto the Fe_3_O_4_@SiO_2_-NH_2_ surface.

### 3.3. Morphological Analysis

**Figure 3:**
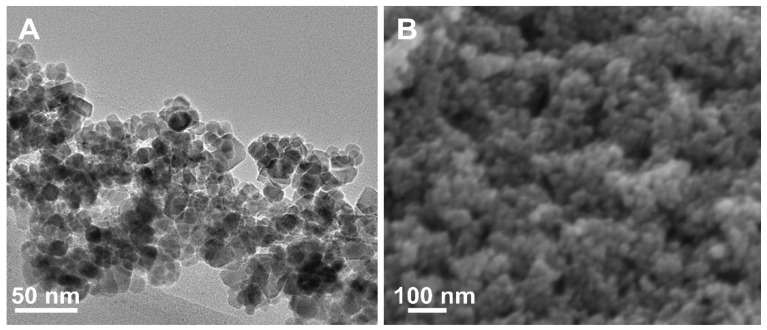
(**A**) TEM and (**B**) SEM images of Fe_3_O_4_@SiO_2_-TbDPA.

The morphology of the as-prepared Fe_3_O_4_@SiO_2_-TbDPA hybrid material was investigated by transmission electron microscopy (TEM) and scanning electron microscopy (SEM) simultaneously. The TEM image showed that iron oxide nanoparticles were well encapsulated in the SiO_2_ layer (Figure 3A). The SEM graph supported that Fe_3_O_4_@SiO_2_-TbDPA hybrid materials were almost uniform, and regular spheres were found (Figure 3B). According to the analysis, the homogenous Fe_3_O_4_@SiO_2_-TbDPA nanospheres were established.

### 3.4. Magnetic Properties

**Figure 4:**
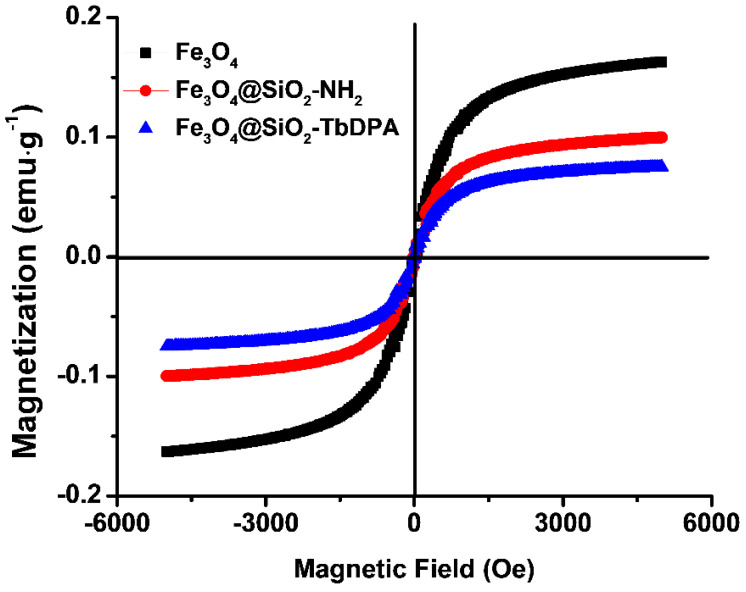
Magnetic hysteresis loops of the Fe_3_O_4,_ Fe_3_O_4_@SiO_2_-NH_2_ and Fe_3_O_4_@SiO_2_-TbDPA.

Magnetic properties of Fe_3_O_4_, Fe_3_O_4_@SiO_2_-NH_2_ and Fe_3_O_4_@SiO_2_-TbDPA nanospheres were investigated at room temperature by vibrating sample magnetometer (VSM) in the field range from −5000 to 5000 Oe (Figure 4). The magnetization curves represent the soft magnetic behavior of the ferrite samples, which is beneficial for improving EM wave absorption [39]. The values of remanent magnetization (Mr), saturation magnetization (Ms) and coercivity (Hc) at room temperature were provided in Table S1. Compared with free Fe_3_O_4_ NPs (0.163 emu·g^−1^), the functionalized magnetic Fe_3_O_4_@SiO_2_-NH_2_ nanospheres were lower and had a magnetization saturation value of 0.099 emu·g^−1^. Such reduction in magnetism could be mainly attributed to the non-magnetic SiO_2_ layer coating on the Fe_3_O_4_ nanoparticles’ surface. Similarly, the saturation magnetization of the Fe_3_O_4_@SiO_2_-TbDPA magnetic nanospheres was found to be 0.075 emu g^−1^. After grafting DPA, the magnetic properties of Fe_3_O_4_@SiO_2_-NH_2_ were further decreased. In addition, the Hc values of the Fe_3_O_4_, Fe_3_O_4_@SiO_2_-NH_2_ and Fe_3_O_4_@SiO_2_-TbDPA nanocomposites were 33.856, 26.332 and 18.809 Oe, respectively. The low coercivity could be ascribed to the low resonance frequency [40,41]. Fortunately, the Fe_3_O_4_@SiO_2_-TbDPA nanospheres were easily separable under exposure to an external magnetic field, which proved that these magnetic nanomaterials possessed excellent magnetic properties and could be used for potential applications.

### 3.5. Selective and Sensitive Detecting NO_2_^−^

**Figure 5:**
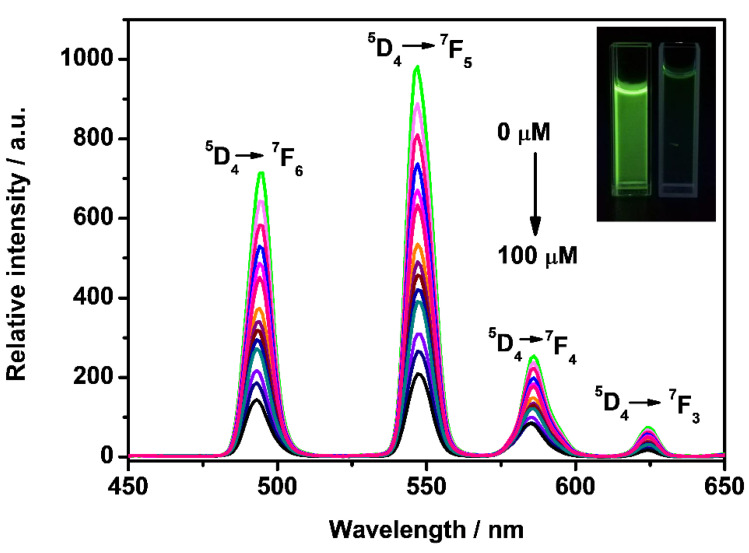
Emission spectra of DPA-Fe_3_O_4_@SiO_2_-Tb (0.1 mg/mL) aqueous solution upon the addition of NO_2_^−^ (0–100 μM) under 290 nm excitation. (Inset: photographs of Fe_3_O_4_@SiO_2_-TbDPA dispersions taken before (left) and after (right) the addition of 100 µM NO_2_^−^ under 254 nm UV lamp).

**Figure 6:**
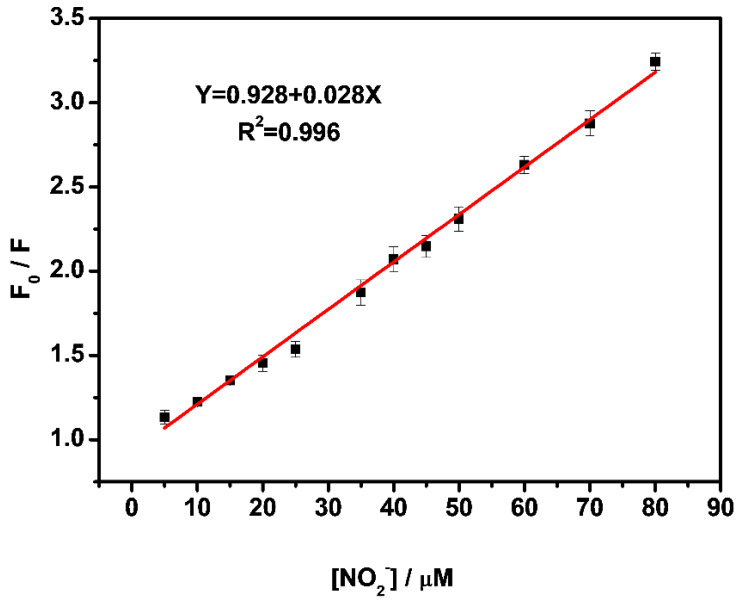
Linearity of fluorescence intensity ratio (F_0_/F) of Fe_3_O_4_@SiO_2_-TbDPA versus NO_2_^−^ ion concentration (F_0_ and F represent the fluorescence intensities at 546 nm in the absence and presence of nitrite, respectively).

To verify the fluorescence properties of Fe_3_O_4_@SiO_2_-TbDPA, the excitation and emission spectra were recorded. The excitation spectrum is dominated by the peaks centered at 249 nm and 290 nm, which were identified by monitoring the emission of Tb(III) ions at 546 nm (Figure S2). In its emission spectrum, the Tb(III) ion signal was evident from the appearance of linear emission bands at 495, 546, 586, and 624 nm, respectively, corresponding to the deactivation of the Tb(III) excited states ^5^D_4_ → ^7^F_6_, ^5^D_4_ → ^7^F_5_, ^5^D_4_ → ^7^F_4_, and ^5^D_4_ → ^7^F_3_ (excited wavelength at 290 nm). Under the irradiation at 254 nm UV light, its characteristic green emission was observed with the naked eye (insert photo in Figure 5). Upon the addition of various concentrations NO_2_^−^ (0 μM to 100 μM), the fluorescence intensity of Fe_3_O_4_@SiO_2_-TbDPA gradually decreased and eventually almost disappeared (Figure 5). The fluorescence intensity variation of Fe_3_O_4_@SiO_2_-TbDPA versus the concentration of NO_2_^−^ followed the excellent linear equation Y = 0.928 + 0.028X (R^2^ = 0.996) (Figure 6). The detection of limit (DL) was determined to be 1.03 μM according to the equation DL = 3 × SD/slope, where SD was the standard deviation of the blank sample. The calculated DL is much lower than the MCL of NO_2_^−^ ions in drinking water permitted by WHO and EPA. The detection limit in our proposed method has been compared with various published literature (Table S2). It is believed that the magnetic Fe_3_O_4_@SiO_2_-TbDPA nanoprobes provide acceptable values in terms of detection limits and allow assays in 100% aqueous solutions [5,7,32,42,43,44,45,46,47]. Moreover, the magnetic Fe_3_O_4_@SiO_2_-TbDPA nanoprobes can be separated from the aqueous solution by taking advantage of their magnetic properties (Figure S3). The proposed method has the unique advantages of simple operation, high selectivity, high sensitivity and low cost.

**Figure 7:**
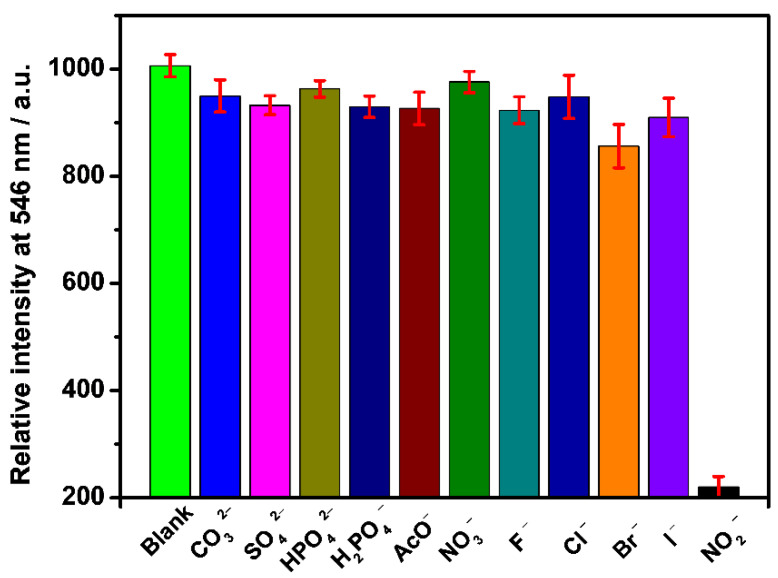
Fluorescence intensity of Fe_3_O_4_@SiO_2_-TbDPA (0.1 mg/mL) aqueous solution at 546 nm in the presence of 100 μM (CO_3_^2−^, SO_4_^2−^, HPO_4_^2−^, H_2_PO_4_^−^, AcO^−^, NO_3_^−^, F^−^, Cl^−^, Br^−^, I^−^ and NO_2_^−^), respectively.

Selectivity is an important index for evaluating fluorescent probes [48,49,50]. To explore the selectivity performance of Fe_3_O_4_@SiO_2_-TbDPA, we performed analogous experiments upon the addition of 100 μM of CO_3_^2−^, SO_4_^2−^, HPO_4_^2−^, H_2_PO_4_^−^, AcO^−^, NO_3_^−^, F^−^, Cl^−^, Br^−^ and I^−^. No obvious changes were detected except NO_2_^−^ (Figure 7). NO_2_^−^ is a selective quencher for Tb^3+^ luminescence. This is due to the interaction between NO_2_^−^ and Tb^3+^, and the fluorescence quenching is attributed to the energy transfer from Tb^3+^ to NO_2_^−^. These results supported the selectivity of the Fe_3_O_4_@SiO_2_-TbDPA nanoprobe for the effective recognition of NO_2_^−^ in aqueous solutions. Overall, this magnetic nanoprobe exhibited great potential in the recognition of NO_2_^−^.

### 3.6. Detection of Nitrite Ions in Tap Water Sample

To investigate the practical applicability of the nanoprobe Fe_3_O_4_@SiO_2_-TbDPA, we have measured the emission intensity of tap water with varied NO_2_^−^ concentrations. Appropriate amounts of NO_2_^−^ (5, 10, 30 and 50 μM) were added to the tap water, and the final NO_2_^−^ content was measured in all samples (4.75, 10.9, 28.9 and 50.9 μM) (Table S3). The average recoveries of nitrite for all spiked samples were in the range of 96–108%, and low relative standard deviations (2.2–3.1%) were obtained, which would be sufficient for practical use. These results substantiated that the proposed determination strategy could be valuable in real samples, suggesting its possibility in the sensing field and analytical assays.

## 4. Conclusions

In conclusion, we have successfully designed and synthesized a novel water-dispersible Fe_3_O_4_@SiO_2_-TbDPA inorganic-organic hybrid nanoprobe for the rapid and sensitive detection of NO_2_^−^ in 100% aqueous solutions. The as-prepared nanoprobe exhibited a good linear response for NO_2_^−^ concentrations from 5 μM to 80 μM with a lower limit of detection (1.03 μM). After detection, the magnetically sensitive nanoprobe Fe_3_O_4_@SiO_2_-TbDPA could be effectively separated from the aqueous solution using its magnetic properties. Therefore, this novel nanoprobe Fe_3_O_4_@SiO_2_-TbDPA can provide a promising way for NO_2_^−^ measurements under practical conditions.

## Data Availability

The data that support the findings of this study are available from the corresponding author, upon reasonable request.

## Supplementary Materials

The following supporting information can be downloaded at: https://www.mdpi.com/article/10.3390/molecules27144431/s1, Figure S1: UV-Vis spectra of Fe_3_O_4_, Fe_3_O_4_@SiO_2_-NH_2_ and Fe_3_O_4_@SiO_2_-TbDPA in aqueous solution; Figure S2: Emission spectra of Fe_3_O_4_@SiO_2_-TbDPA (0.1 mg/mL) in aqueous solution; Figure S3: Photograph of a magnet attracting Fe_3_O_4_@SiO_2_-TbDPA in aqueous solution; Table S1: Magnetic parameters of Fe_3_O_4_, Fe_3_O_4_@SiO_2_-NH_2_ and Fe_3_O_4_@SiO_2_-TbDPA nanocomposite; Table S2: Comparison between the current method and the reported literatures for the detection of nitrite; Table S3: Results of determination of nitrite in tap water (*n* = 3).

## Sample Availability

Samples of the compounds are not available from the authors.
