# Supplementary material for: Lanthanide Molecular Species Generated Fe3O4@SiO2-TbDPA Nanosphere for the Efficient Determination of Nitrite
Source: Molecules. 2022 Jul 11;27(14):4431. doi: 10.3390/molecules27144431 (PMC9315872; doi:10.3390/molecules27144431)
Supplement: Supplementary file 1 [file molecules-27-04431-s001.zip › molecules-1801689-supplementary.pdf]

molecules

Supplementary Materials

Lanthanide Molecular Species Generated Fe_3O_4@SiO_2-TbDPA Nanosphere for the Efficient Determination of Nitrite

Xiangqian Li [1,2], Qin Wen [3], Jiannian Chen [2], Wenjie Sun [2], Yuhui Zheng [2], Chenggang Long [4] and Qianming Wang [2,*]

1 Key Lab of Ecological Restoration in Hilly Areas, School of Chemical & Environmental Engineering, Pingdingshan University, Pingdingshan 467000, China; lixq605@163.com
2 Guangzhou Key Laboratory of Analytical Chemistry for Biomedicine, School of Chemistry, South China Normal University, Guangzhou 510006, China; a18925131809@163.com (J.C.); 13842933314@163.com (W.S.); yhzheng78@scnu.edu.cn (Y.Z.)
3 Institute of Biomedical Engineering, College of Life Sciences, Qingdao University, Qingdao 266071, China; qwen@qdu.edu.cn
4 Ruide Technologies (Foshan) Inc. Foshan, Guangdong 528311, China; hychen7788@126.com
* Correspondence: qmwang@scnu.edu.cn; Tel.: +86-20-39310258; Fax: +86-20-39310187

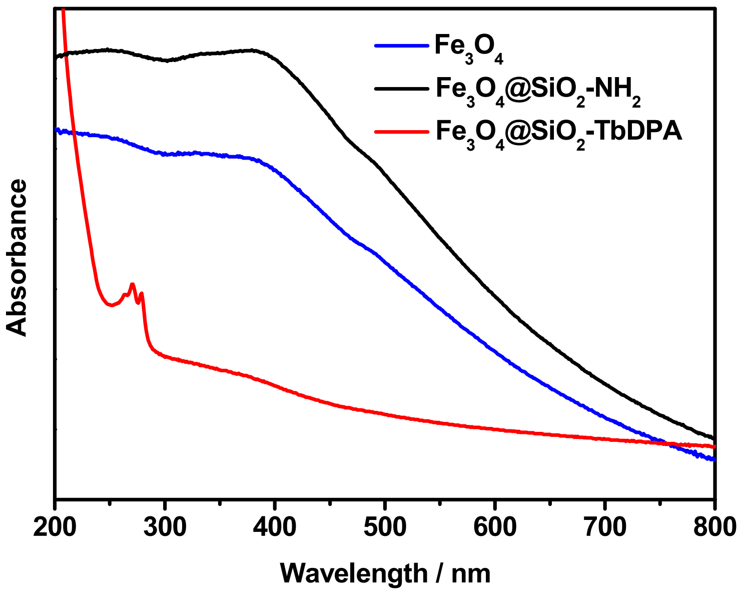

Figure S1. UV-Vis spectra of Fe_3O_4, Fe_3O_4@SiO_2-NH_2 and Fe_3O_4@SiO_2-TbDPA in aqueous solution.

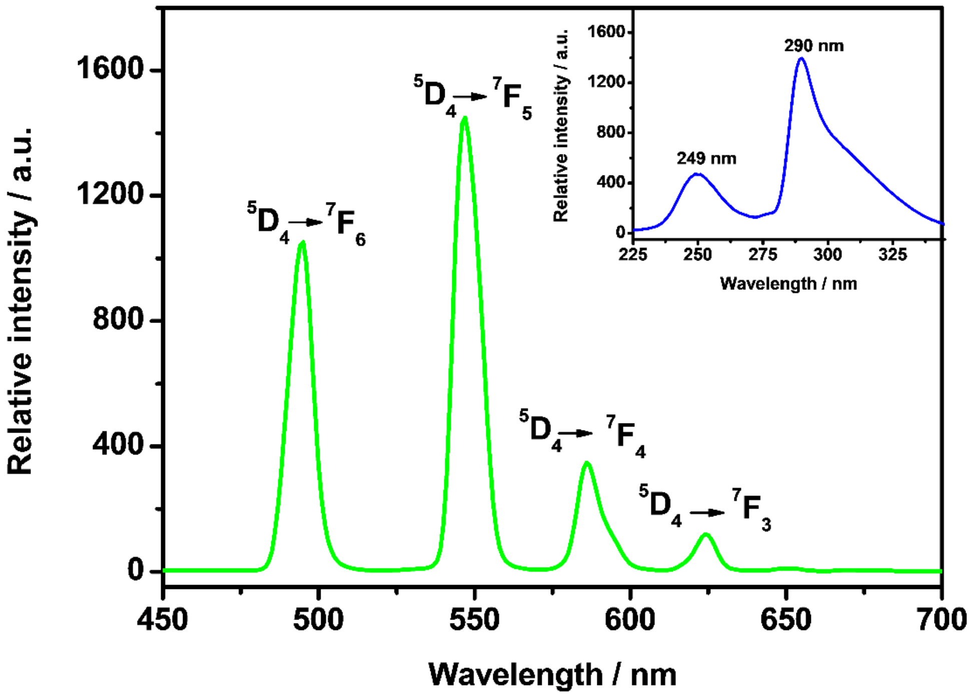

Figure S2. Emission spectra of $Fe_3O_4@SiO_2$-TbDPA (0.1 mg/mL) in aqueous solution. (Inset: Excitation spectrum of $Fe_3O_4@SiO_2$-TbDPA) (emission wavelength at 546 nm).

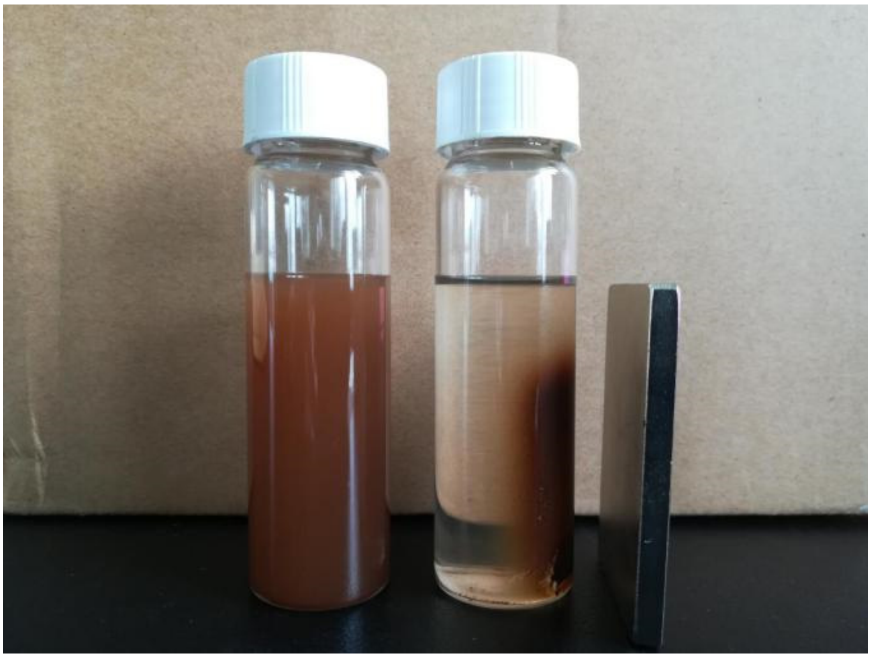

Figure S3. Photograph of a magnet attracting $Fe_3O_4@SiO_2$-TbDPA in aqueous solution.

Table S1. Magnetic parameters of Fe_3O_4, Fe_3O_4@SiO_2-NH_2 and Fe_3O_4@SiO_2-TbDPA nanocomposite.

Samples	Ms(emu/g)	Hc(Oe)	Mr(emu/g)
Fe_3O_4	0.163	33.856	0.00453
Fe_3O_4@SiO_2-NH_2	0.099	26.332	0.00691
Fe_3O_4@SiO_2-TbDPA	0.075	18.809	0.00477

Table S2. Comparison between the current method and the reported literatures for the detection of nitrite.

Method	Materials	Detection limit	References
Surface-enhanced Raman spectroscopy	Fe_3O_4@SiO_2/Au	13.69 μM	[5]
Electrochemical	SiO_2/C/MnPc	0.79 μM	[7]
Electrochemical	Fe_3O_4-MWNTs	1.19 μM	[32]
Fluorescence	Fe_3O_4@ MCM-41	1.1 μM	[42]
Fluorescence	Fe_3O_4@Rh 6G	0.8 μM	[43]
Fluorescence	Carbon dots	0.65 μM	[44]
Fluorescence	Graphene quantum dot	9.8 μM	[45]
Fluorescence	Carbon dots	30 μM	[46]
Fluorescence	UiO-66-NH_2	77 μM	[47]
Fluorescence	Fe_3O_4@SiO_2-DPA	1.03 μM	This work

Table S3. Results of determination of nitrite in tap water (n=3).

Sample number (No.)	Nitrite added (μM)	Nitrite found (μM)	Recovery (%)	RSD (%)
No. 1	5	4.8	96	2.2
No. 2	10	10.8	108	2.7
No. 3	30	29.1	97	3.1
No. 4	50	50.5	101	2.8
